# A Hybrid Spider Monkey and Hierarchical Particle Swarm Optimization Approach for Intrusion Detection on Internet of Things

**DOI:** 10.3390/s22218566

**Published:** 2022-11-07

**Authors:** Sandhya Ethala, Annapurani Kumarappan

**Affiliations:** Department of Networking and Communications, SRM Institute of Science and Technology (SRMIST), Kattankulathur Campus, Kattankulathur 603203, India

**Keywords:** hierarchical particle swarm optimization, internet of things, intrusion detection system, random forest classifier, spider monkey optimization

## Abstract

The Internet of Things (IoT) network integrates physical objects such as sensors, networks, and electronics with software to collect and exchange data. Physical objects with a unique IP address communicate with external entities over the internet to exchange data in the network. Due to a lack of security measures, these network entities are vulnerable to severe attacks. To address this, an efficient security mechanism for dealing with the threat and detecting attacks is necessary. The proposed hybrid optimization approach combines Spider Monkey Optimization (SMO) and Hierarchical Particle Swarm Optimization (HPSO) to handle the huge amount of intrusion data classification problems and improve detection accuracy by minimizing false alarm rates. After finding the best optimum values, the Random Forest Classifier (RFC) was used to classify attacks from the NSL-KDD and UNSW-NB 15 datasets. The SVM model obtained accuracy of 91.82%, DT of 98.99%, and RFC of 99.13%, and the proposed model obtained 99.175% for the NSL-KDD dataset. Similarly, SVM obtained accuracy of 85.88%, DT of 88.87%, RFC of 91.65%, and the proposed model obtained 99.18% for the UNSW NB-15 dataset. The proposed model achieved accuracy of 99.175% for the NSL-KDD dataset which is higher than the state-of-the-art techniques such as DNN of 97.72% and Ensemble Learning at 85.2%.

## 1. Introduction

**Figure 1:**
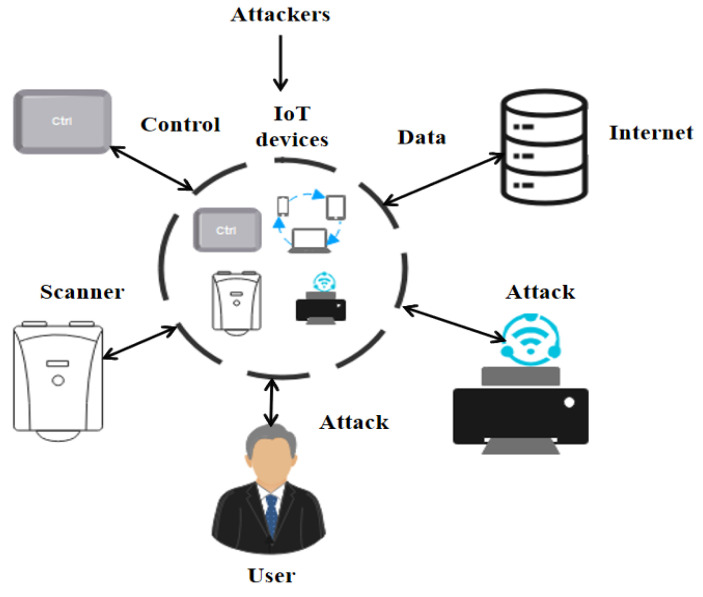
Pictorial Representation of attackers towards IoT devices.

The Internet of Things (IoT) provides services for different fields for data exchange in the infrastructure [1]. There are various applications of IoT, including smart homes, smart cities, hospitals, supply chain system, smart grid systems, intelligent transportation systems, etc. The IoT includes embedded sensor networks, physical objects, electronics, software connection, and embedded electronics to exchange the data [2]. The IoT connection helps significantly with data exchange, improving human lives, and thus, the crucial part of the IoT is the cyber security problem, which affects the development of applications [3]. The IoT applications are more vulnerable to the malicious agents, so the IoT devices for processing have reduced capacity. As the number of IoT devices grows, there is a greater risk of security vulnerabilities, such as data theft and the possibility of Distributed Denial of Service (DDoS) attacks [4]. The DDoS attacks will usually damage the node availability via signal jamming or resource exhausting in the node’s battery. There are two types of DDoS attack. One crashes the services and other floods the services [5]. The Denial of Service (DoS) characterizes the attackers based on an explicit attempt at services prevention that deploys the IoT devices to reach the goals. The server is compromised when the attacks occur, and the IoT devices of the network are affected, resulting in an industrial loss [6]. There are various attacks present which target IoT devices, such Mirai, Hajime, Hide and Seek, Bashlite, Tsunami, Brickerbot, and Luabot. There have been various attacks initiated in the computer networks such as Port Scanning, Brute Force, Remote to Local (R2L), Denial of Service (DoS), User to Root (U2R), and Probing, etc. Figure 1 shows the representations of IoT towards the attackers. Network-based Intrusion Detection System (NIDS) are useful for monitoring the network infrastructure, and malicious activities are detected [7,8,9]. The open literature is studied when an intruder launches a network in which the intruder has the potential to determine the attacks that use system vulnerabilities. They gain unauthorized access to users’ information in order to take the system down.

Various Machine Learning algorithms are applied to perform NIDS such as Artificial Neural Networks (ANN) [10], K-Nearest Neighbor (KNN) [11], Naive Bayesian (NB), and Support Vector Machine (SVM) [12]. However, the algorithms require the data to process the missing values of unstable results, and they run slowly because of the large amount of data. Therefore, optimization methods such as Particle Swarm Optimization (PSO), Whale Optimization Algorithm (WOA), Dolphin Swarm Algorithm (DSA), Gray Wolf Optimization (GWO), etc., were used to select the appropriate features for IoT attacks classification in the environment. Feature selection was used to discard the redundant and irrelevant features and choose the optimal feature subset to improve the pattern definition that belongs to dissimilar classes [13,14,15]. The extraction of appropriate features was used to minimize the amount of training and guarantee accuracy during the detection [16]. A cooperative classification model was developed by choosing a top set of attributes using the feature selection [17,18]. The prior models did not provide effective feature representation and did not extract the features required to obtain the random Fourier feature needed to fit the linear classifier. The changes in the large size of data and the unnecessary changes increased the operating time; hence, the desired results were not achieved during the intrusion detection.

From the literature, it is concluded that various security challenges and issues were present in the IoT application. The key challenges faced are as follows:

- Designing a security framework for the IoT application is a problematic task because of the dynamic IoT network nature.
- The security mechanism for IoT is ensured by using an appropriate tool for distribution of architecture, which has the ability to analyze a huge amount of data. This was a challenge.
- The network environment has interconnected devices, which includes poorly designed sensors and authentication measures.

Therefore, the above issues are addressed by using a Hybrid SMO-HPSO that analyzed the huge amount of data via IoT devices. The IDS has the advantages of mitigating the attacks in IoT and ensures the security mechanism by using an appropriate tool. The proposed method uses the hybrid feature selection algorithm that identified the appropriate features for discarding the irrelevant features. Therefore, the hybrid feature selection is used to obtain the subset of features that results with improvement in the performances. The RF classifier is employed to classify and identifies the attacks as normal and abnormal attack observations.

Thus, the main contributions of this study are mentioned as follows:

- The hybrid feature selection that combines SMO and HPSO is developed to solve the massive intrusion data classification problem and improve the accuracy rate of attack detection. The local searching phase of conventional SMO is operated in a random manner, which affects the optimal score value. In order to overcome this issue, the hierarchical PSO’s velocity is combined with SMO’s searching process to obtain the optimal feature subset.
- Further, the feature importance with Rosenbrock function is included as gradient for Hybrid SMO-HPSO. Rosenbrock is a parabolic mathematical function which represents the probability density function of any events. In this research, the probability of features contributing to the target class is defined by the Rosenbrock function, so the Hybrid SMO-HPSO does not require any classified instance output to compute the fitness.
- To enhance the accuracy that shows lower classification error, the attacks for NSL-KDD are classified into four types of attack, including DoS, U2R, R2L, and probing attack. The UNSW Dataset classifies the attacks into normal and abnormal classes.

The structure of this study is as follows: Section 2 examines related studies of IDS detection for IoT attack categorization. Section 3 elaborates on the methodology used for the development of the hybrid model. Section 4 presents a simulation comparison and performance evaluation to demonstrate the efficacy of the suggested model. The conclusion is given in Section 5.

## 2. Related Works

In the field of IDS, many scholars have conducted various research studies using a machine learning algorithm by using publicly available datasets. The main aim of the existing models was to improve the intrusion detection rate.

### 2.1. Attack Detection Based on Classification

Su et al. [12] created a Bidirectional Long Short-term Memory and Attention Mechanism (BAT) to detect network intrusions. The created model was capable of automatically learning the hierarchy’s essential characteristics. The Attention mechanism was used to filter the network flow vector, which was made up of packet vectors derived by BLSTM. The primary characteristics produced by the BLSTM model were utilized to classify network traffic. Multiple convolution layers were used to extract local characteristics from traffic data. The data samples were processed, and the BAT model classifies them using the Softmax classifier. The created technique has difficulties selecting features and cannot effectively handle the huge intrusion data classification, which results in low identification accuracy and a high false alarm rate.

Gao et al. [19] created an adaptable Ensemble Machine Learning (EML) model for intrusion detection. Using an adaptive ensemble learning approach, the created model considered the NSL-KDD dataset for the research. The multi tree technique was built using training data and the setup of several decision trees. Classifiers such as Decision Tree, Random Forest, k-Nearest Neighbor (k-NN), Deep Neural Network (DNN), and developing an EML adaptive voting algorithm were utilized to increase the overall detection impact. The preprocessing module converts all character-type information, such as label and service, to numbers, standardizes the data, and removes superfluous features. However, the proposed technique was not centralized sufficiently to show difficulties with categorization because R2L and U2R data were overlapped with other data.

Çavuşoğlu [20] created a hybrid of machine learning and feature selection approaches such as Artificial Neural Network (ANN) and Support Vector Machine (SVM). The proposed model initially performs data preprocessing on the NSL-KDD dataset by means of multiple feature selection techniques, after which the dataset size was decreased. For the feature selection procedure, the existing two techniques were used. Based on the type of attack, the layered architecture selects the relevant machine learning algorithms. It was able to create an effective result for lesser attributes from the dataset based on the feature selection approaches. On the basis of protocol type, the most significant components used showed network traffic. However, unnecessary and data analysis were employed on huge datasets, resulting in longer processing times and failure to reach the intended performance.

Wu et al. [21] created an Improved DBN (IDBN) using feature weighted SVM for intrusion detection. An adaptive learning rate method was utilized to improve the training performance of the IDBN, which was used to learn deep features from raw data to reduce dimensionality. Second, the PSO method was used to optimize the SVM, which was followed by determining the weights of deep features and the optimal Gaussian kernel parameters. This results in the WSVM, which is used to remove weakly linked and redundant features from the IDBN-derived features. The IDBN-WSVM model was validated using the NSL-KDD dataset, which decreased the training time. The testing time for the large-scale datasets was more resilient and had a higher generalization performance. The presented technique did not demonstrate effective feature representation and failed to extract features to generate random Fourier features to suit a linear classifier.

Alternatively, various research studies were presented which consisted of articles pointing out the success using feature selection algorithms in NIDS. Kumar et al. [22] developed an integrated rule-based IDS system for the classification of the attacks. In the existing IDS techniques, the anomaly-based model classifies for both parts of the combination. Additionally, the developed model showed improvement in the performances when the developed model was integrated to perform a classification-based model that detected the five categories using the UNSW-NB 15 dataset. However, an enhancement in detection is required for real time data to classify new unknown attacks.

Ramaiah et al. [23] utilized an Optimized Deep Neural Network (ODNN) for ID. The existing models showed challenges in aspects of network security. The ID is important, as it effectively mitigates the organization vulnerability. The IDS uses a correlation tool and a random forest to detect the independent variables predominantly for neural-based network classification. The models detected the malicious attacks using a shallow neural network and an optimization approach, which used a shallow neural network and an optimized neural-based classifier. The results obtained during the experiment showed that the proposed IDS was higher for the counterpart intrusion detection that needed improvement in memory for the complex neural networks.

Ajdani and Ghaffary [24] designed a Support Vector Machine (SVM) for IDS. The developed model designed an analytical framework to detect the destructive data using three factors: users’ information, scale, and time. The developed model trained the data and divided them into sub periods which exploited user review information at each time period when the data were trained. The storage methods were applied to enhance the scalability in terms of speed, which reduced the computation volume. However, the scalability factor was increased, as the model had used external search and destructive data continuously.

Hsu et al. [25] used LSTM-based CNN for network intrusion detection. The problem was overcome by the deep learning schemes, as it provided the solution for several fields. The deep learning model is constructed using CNN-LSTM layers, which classify each part of the traffic network. The proposed deep learning model is constructed on the basis of Convolutional Neural Network (CNN), with LSTM achieving well in various fields. However, an extra model is required to be implemented in a real-time traffic network.

Kasongo and Sun [26] created IDS using Feed Forward Deep Neural Networks (FFDNNs) and a filter-based feature selection method. The technique produced optimum subsets of characteristics with the least amount of redundancy. The IDS system created was based on deep learning with FFDNNs and a filter-based feature selection method. The FFDNN-IDS was tested on the well-known NSL-KDD dataset and compared against current classifiers, such as SVM and Naïve Bayes. The majority of misclassifications occurred because they were uncommon in both the training and test datasets.

### 2.2. Attack Detection Based on Optimization Algorithms

The previous section discussed the classification; however, processing of data is needed for the missing values, as the algorithms used were unstable. Therefore, optimization algorithms were used to select the appropriate features for the IoT attacks classification in the environment. The existing models that were involved in attack detection using optimization algorithms are as follows:

The feature selection algorithm effectively influenced the ML-based IDS model that explained the feature selection and classification under the training phase. Elmasry et al. [11] established a deep learning approach for the detection of intrusions in the network by utilizing a double Particle Swarm Optimization (PSO) algorithm. The optimum features were selected from datasets such as NSL-KDD and CICIDS2017 datasets to perform the binary and multiclass classification tasks. The pretrained phase was used for the selection of optimized features taking care of the automated hyper parameters. The upper level and the optimized hyper parameters were estimated. They increased the accuracy by reducing the datasets because of double PSO. The deep learning model Deep Belief Networks (DBN) was utilized to investigate the performance differences during classification. However, the developed approach showed a drawback that increases the time needed for training and testing, which was not acceptable for all scenarios. Several studies were suggested by the selection of relevant features for IDS that showed possibilities by considering improvement in detection of performance and accuracy.

Jiang et al. [27] developed a PSO-Xgboost for IDS networks. The Improved model demonstrated NIDS in detection accuracy. The existing PSO-Xgboost model generated a classification model using Xgboost, and then PSO was used to adaptively search for the best structure of Xgboost. The developed model suggested the model’s performance; the created model employed the NSL-KDD dataset and showed improvement in the results. The investigational outcomes show that the PSO-Xgboost outperforms in detecting minority attack groups, such as U2R and R2L. However, when the iterations for the number of particles is minimal and the algorithm falls under the local optimum solution, it lowers the feature selection probabilities.

Alazzam et al. [28] developed a feature selection algorithm for IDS based on Pigeon Inspired Optimizer (PIO). The developed algorithm performed the feature selection which depends on pigeon behavior. The developed model utilized KDDCUP99, NSL-KDD and UNSW-NB15 datasets for the evaluation of the results. The developed model performed a discretization process to obtain a continuous swarm intelligent algorithm for solving the problem. The developed model used the cosine similarity to find the fitness function. The binaries processing limited the landmark operator effectiveness that lowered the accuracy measures. Thus, the cyber-attacks mitigate the attacks in IoT devices and the present research uses IDS in the IoT environment.

## 3. Proposed Methodology

**Figure 2:**
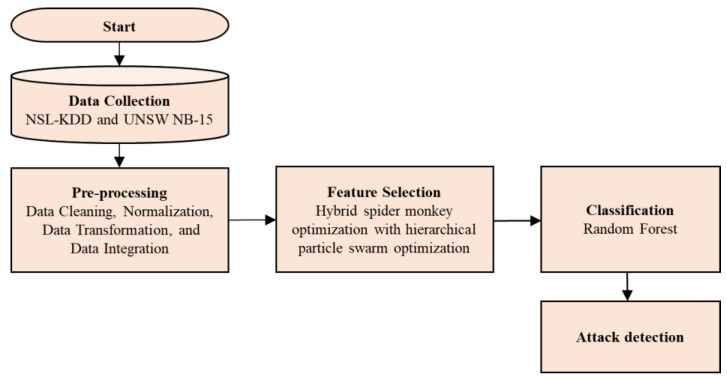
The block diagram of proposed SMO-HPSO feature selection method.

NSL-KDD and UNSW NB-15 are the datasets utilized in this research work. The unwanted data are eliminated in the datasets after data preprocessing has been performed. In SMO-HPSO, SMO deliberate the features from which the leader determines whether to divide or merge, resulting in the optimum feature value being selected. Similarly, HPSO will organize the particles hierarchically depending on their best function and then use RFC to classify the attacks. The flow chart of SMO-HPSO is presented in Figure 2.

### 3.1. Data Collection

The NSL-KDD dataset is applied to the IoT context, which is based on the original KDD Cup 99 datasets that are obtained by removing the duplications and redundancies presented in them. The NSL-KDD dataset has 41 labeled characteristics, which include attack types. The NSL-KDD is proposed to alleviate the issues occurring in the KDD’99 datasets [29]. The training and test sets consist of a reasonable number of recordings. This benefit makes it possible to perform the tests on the entire collection without having to randomly choose a tiny piece. As a consequence, the assessment outcomes of various research projects will be uniform and equivalent.

The NSL-KDD has four attacks, which are outlined as follows: DoS, U2R, R2L, and probing Attack.

Probe attack: 

During the scanning process, the probe attack occurs, which involved gathering abuse of data and information from the network.

R2L: The packets acquired are sent and computer detects the flaws present in the network.

U2R: Once the ordinary account is obtained, U2R has access to the root account. Load module, Buffer overflow, Xterm, Rootkit, Perl, Ps, and Sql attack are some of the assaults in U2R.

DoS: Due to increased network traffic use, the system is unable to offer a service, resulting in a DoS attack. DoS attacks include Back, Apache2, Udp Storm, Smurf, Neptune, Pod, Land, Teardrop, and Worm, all of which which can steal data via the internet.

The dataset consists of 53,873 records for KDD Train+ for training and validation of the subset. It has 6735 normal records, and 6735 for the test subset which contains anomalous data. 

In addition, the UNSW NB-15 dataset is utilized to evaluate the suggested technique, which is made up of 15 datasets generated based on the IXIA Perfect Storm tool. The Cyber Range Lab of University of New South Wales (UNSW), Canberra, generates a mix of actual and regular activities for identifying attack behaviors. The dataset comprises nine different attacks, including backdoors and denial-of-service attacks. Generic, Exploits, Generic, Shellcode, Reconnaissance, Fuzzers, and Worms are also examples of generics. The Argus and Bro-IDS are exploited in 12 methods to create a total of 49 class-labeled features. The Argus and Bro-IDS tools generate them using 12 methods, and a total of 540,044 records are saved in CSV files. From the UNSW NB-1515 GT.csv, the ground truth images are taken. The event list is known as the ground truth, also called UNSW NB-1515 GT.csv, where the event list is called UNSW-NB15 LIST EVENTS.csv. For training and testing, the partition is taken from the configured dataset. There are a total of 175,341 training set records and 82,332 testing set records from different sorts of attacks from regular attacks [30]. The information of UNSW and NSL-KDD dataset is provided in Table 1, which includes statistical information.

### 3.2. Preprocessing

Several data preprocessing methods are used, including data cleaning, normalization, and data integration. The data cleaning process involves actions such as removing duplicated data points, standardized datasets, and syntax errors, as well as missing codes, empty fields, and finding missing codes. The cleaning of the data from the datasets is standardized to perform data analysis and it can be conveniently accessed for finding the appropriate data. In normalization, data vectors are introduced as new vectors. The key advantages of data normalization are enhanced consistency and a reduced risk of data inconsistency. The Min–Max normalization model is critical for the integration and normalization process. Every feature value has minimum value that converts to zero and one as the maximum value. The normalisation procedure is expressed by Equation (1).

(1) $$X_{norm}=\frac{X_{i}-X_{min}}{X_{max}-X_{min}}$$

where $X_{i}$ is the data point, $X_{min}$ and $X_{max}$ is referred as minimum and maximum value of the data point with batch instances. The main importance of Recursive Feature Elimination (RFE) is that it provides importance values by ranking the most important features. It is supposed that data redundancy is eliminated and yields more compact feature subsets. The features are arranged from high importance to low importance, and thus the ranking of features will be obtained.

### 3.3. Feature Selection

**Figure 3:**
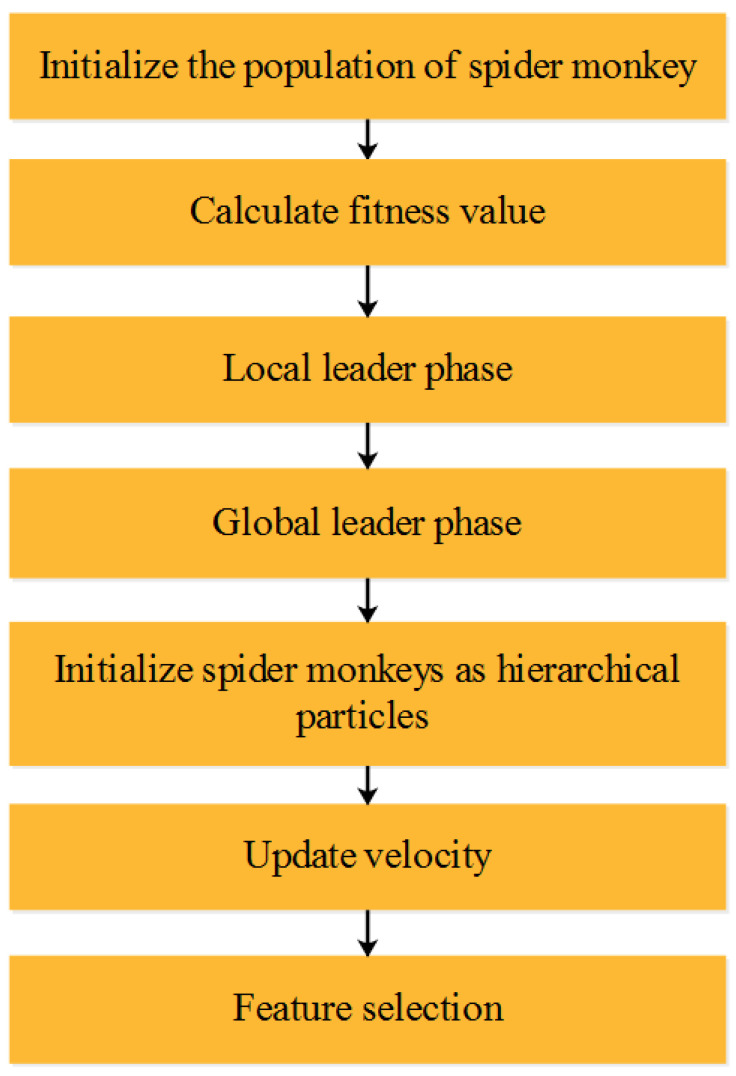
Steps involved in hybrid feature selection algorithm.

After the data have been extracted, the feature selection process occurs to detect low-rate attacks using a hybrid method combining SMO and H-PSO. The hybrid algorithm enhances the accuracy and includes lower classification error, and classifies the attacks among Probe, DoS, R2L, and U2R. The outcomes are verified using various performance measures that are explained in the next section. In Hierarchical PSO (H-PSO), the motion of H-PSO is affected by three aspects: movement induced by swarm individuals, foraging activity, and physical diffusion motion, which are explained using the flow chart in Figure 3. The HPSO is known as the meta-heuristic technique that is similar to Spider Monkeys (SM). The SMs activities are found on social formation and foraging behaviour. The fission–fusion features are extracted based on the algorithm where the leader is from the group dependent on social organisation.

#### 3.3.1. Spider Monkey Optimisation Algorithm

SMO is an algorithm that is designed based on the swarm intelligence which has a small group of SMs that are inspired by an intelligent foraging SMs behavior. The fission is created for single group that contains fewer monkeys, which defines the time for fusion. The algorithm is dependent on the social organization of a group of features from the leader, which makes a decision whether to combine or split to search for food. The leader of overall groups is the global leader and leader for each group is the local leader.

#### 3.3.2. Particle Swarm Optimization

The social activities are stimulated through the fundamental and primary behavior of fish schooling and bird flocking. The food searching is performed for the scattered data or settled on the position based on the place where the food is found. The food is searched for by the agents that move from one place to another, and the agents search the feature subset from its position that moves towards it to find and managing it. The useful messages are transmitted, and the food for the agent is searched from one location to another; thus, it is found. The animals’ behavior calculated on the basis of optimization because of the crowd or swarm is called the particle. For each partner’s position, the PSO algorithm finds it from the crowd and searches for the space globally, and it is updated based on Equations (2) and (3).

(2) $$v_{i}^{k+1}=v_{i}^{k}+c_{1}r_{1}\left(p_{best}-x_{i}^{k}\right)+c_{2}r_{2}\left(g_{best}-x_{i}^{k}\right)$$

(3) $$x_{i}^{k+1}=x_{i}^{k}+v_{i}^{k+1}$$

where, $x_{i}^{k}$ is the current position, $v_{i}^{k}$ is the current velocity for ith particle for kth iteration, $p_{best}-x_{i}^{k}$ is the distance among its current and best position, $g_{best}-x_{i}^{k}\;$ is the distance among the current and global best position, $x_{i}^{k+1}$ is the upcoming position of the particle, $v_{i}^{k}$ is the upcoming velocity of the particle.$\;c_{1}$ and $c_{2}$ are the positive constants, $r_{1}$ and $r_{2}$ are the two random variables ranges between 0 and 1.

#### 3.3.3. Proposed Hybrid Optimization Algorithm

There are various hybridization variants present for the heuristic algorithm. There are two main variants that are low-level and high-level relay or evolutionary techniques which would be heterogeneous of homogeneous techniques used to obtain the solution. The SMO algorithm will represent the potential solutions for SM. The SMO usually comprises six phases, such as Global Leader phase, Local Leader phase, Global Leader Learning phase, Local Leader Decision phase, Leader Learning Phase, and Global Leader Decision phase.

##### Population Initialization

The population P from the SMO is initialized and distributed as 50, which is represented as $SM_{p}\;(where\;p=1,\;2\ldots \;P$). The pth monkey is among the $SM_{p}$ population, and the monkeys present have M dimension vectors. These vectors or the variables overcome the problem as the $SM_{p}$ receives the solution. Thus, the problem option showed by the initialized $SM_{p}$ is expressed as shown in Equation (4):

(4) $$SM_{pq}=SM_{minq}+UR\left(0,\;1\right)\times \left(SM_{maxq}-SM_{minq}\right)$$

where $SM_{pq}$ is known as the pth SM with a qth dimension; $SM_{minq}$ and $SM_{maxq}$ is known as the upper and lower bounds that are moving in the direction of qth; the term q is equal to 1, 2,…M and the random numbers range among $UR\left(0,1\right)$ as it is distributed uniformly that it ranges between [0, 1].

###### Local Leader Phase (LLP)

The SM position is updated as a new position that assigns the fitness value to obtain the higher value, which is compared with the previous locations. The location updated for the lth local group for the pth SM is expressed as shown in Equation (5)

(5) $$SM_{newpq}=SM_{pq}+UR\left(0,\;1\right)\times \left(LL_{lq}-SM_{pq}\right)+UR\left(-1,\;1\right)\times \left(SM_{rq}-SM_{pq}\right)$$

where,$\;LL_{lq}$ is the qth dimension of lth local group leader location; $SM_{rq}$ is the qth dimension, which chooses randomly at the lth local group of SM and r≠p.

###### Global Leader Phase (GLP)

The initialization of LLP is processed, and the SM’s location is computed by using Equation (6). The updating of the location is calculated by using Equation (6):

(6) $$SM_{newpq}=SM_{pq}+UR\left(0,\;1\right)\times \left(GL_{lq}-SM_{pq}\right)+UR\left(-1,\;1\right)\times \left(SM_{rq}-SM_{pq}\right)$$

where GLq is called the GLL, which is the dimension of q=1, 2, 3,… M that selects the indexes arbitrarily. The calculation of fitness function for SM is evaluated based on prbp, which is based on the location of $SM_{p}$, is updated. The proposed method selects a total of 11 features out of 43 features from the NSL-KDD and 11 features out of 49 from UNSW dataset. The candidate’s location is better, and access to the number of possibilities is made better. The proposed result selects a total of 22 features that are relevant to the 92 features processed for feature selection. The 22 features are designated by means of proposed HPSO-SMO, which identifies a features-based 80-20 rule. It finds inputs that most probably have feature importance, which prioritize accessing the possibilities. The working of SMO is based on Rule-based priority, which is expressed as $\underset{i\epsilon S}{\sum }fitness_{feature_{importance}}\geq 0.8$ condition.

From the mentioned condition, S is known as the chosen feature value that is obtained by feeding the data to the tree. The features for attacks classifications are not deliberated. The maximum feature value is obtained for fitness function that is based on the subsequent condition. The probability for the condition calculation is expressed as shown in Equation (7).

(7) $$prbp=f_{n_{p}}\;\sum N_{p}=1f_{n_{p}}\;$$

where, $f_{n_{p}}$ is called the pth SM fitness value, which is the fitness value as N further, and the calculation of new location is calculated using Equation (6). The value obtained is related by the old location, and it is implemented on the basis of fitness value function.

###### Global Leader Learning (GLL) Phase

Under the GLL stage, the selection of features is updated and undergone for the selection using the greedy model. The value of fitness function is selected and generated from the population. The location is based on its optimum value, which assigns the value for the global leader. The update is not performed, and the value is incremented from one to GlobalLimitCount.

###### Local Leader Learning (LLL) Phase

For the local group, the LLL is updated in SM location based on the fitness values from a local group and the location is optimum. It has the value which is assigned for the local leader. A further update is not provided, as it increments to one and will be added with the LLC.

###### Local Leader Decision (LLD) Phase

The leaders from the local groups were fixed and updated the local group is present and the candidates randomly modifies location. From the step 1, the local leader and leader group uses the information using Equation (8)

(8) $$SM_{newpq}=SM_{pq}+UR\left(0,\;1\right)\times \left(GL_{lq}-SM_{pq}\right)+UR\left(0,\;1\right)\times \left(SM_{rq}-LL_{pq}\right)$$

###### Global Leader Decision (GLD) Phase

**Figure 4:**
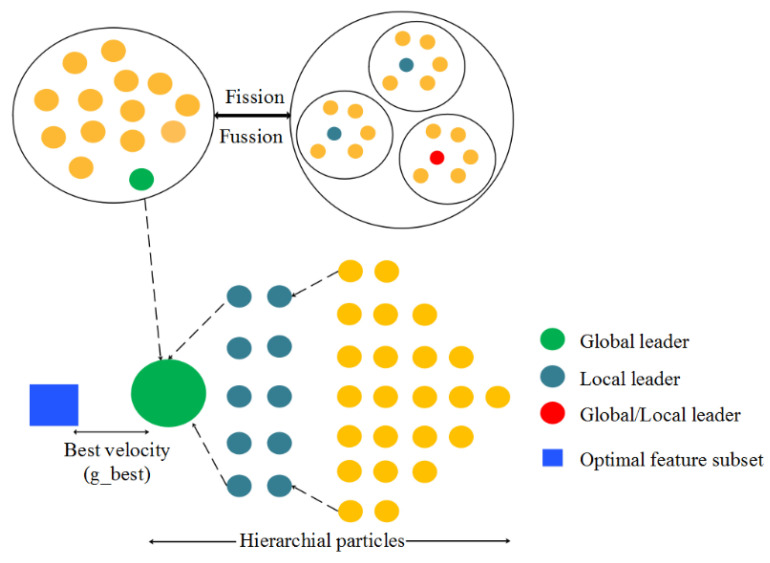
Pictorial representation of the proposed hybrid SMO-HPSO for selecting g-best feature.

The global leader position is not updated due to GlobalLeaderLimit. Populations are split into groups based on the global leader decision. The process of splitting is continued up to the groups until maximum group numbers are reached. For every 10 iterations, the selection of local leader is performed to shape the newly formed group. The groups are allowed at the maximum number to create the global leader, which does not allow a position update unless the boundary decides the entire group from one group. The population from the SMO is based on the actions performed by SMs, which improve the efficiency and deal with the complexity in the real world. In the real world, an optimization algorithm uses a fitness function which is represented pictorially in Figure 4.

The value of fitness is computed using the value of feature importance. This assigns scores for each of the input features on the basis of target variables to perform the prediction. The importance of the feature is computed on the basis of node impurity with the weights, which decreases the probability before the node is reached. The probability for the node is calculated with the ratio of sampled numbers for the overall number of samples. Equation (9) is used to compute the fitness function to select the best features.

(9) $$fitness_{feature_{importance}}=\frac{\;Number\;of\;samples\;that\;reaches\;the\;nodes\;}{\;Total\;number\;of\;samples}$$

The hybridization of SMO with the PSO algorithm generates the hybrid mixed functionality based on the low-level co-evolutionary features. The low-level hybrid functionality features merge and combine variants. The co-evolutionary is used because the variants are used one after the other, running in parallel. The two distinct variants are mixed and are involved in the generation of solutions to overcome the problems. Based on this modification, the hierarchical PSO has the ability to explore SMO to produce the variants with its strength. The PSO and SMO variants are combined, updating the velocity as proposed in Equations (10) and (11)

(10) $$v_{i}^{k+1}=w*\left(v_{i}^{k}+c_{1}r_{1}\left(x_{1}-x_{i}^{k}\right)+c_{2}r_{2}\left(x_{2}-x_{i}^{k}\right)+c_{3}r_{3}\left(x_{3}-x_{i}^{k}\right)\right)$$

(11) $$x_{i}^{k+1}=x_{i}^{k}+v_{i}^{k+1}.$$

The value of the fitness function is utilized to select the best optimum value. The Rosenbrock function, which is known as the objective function, is used in the suggested approach. The Rosenbrock function is efficiently optimized using a suitable coordinate system without exploiting the gradient data, and uses the built-in local without approximation model using Equation (12)

(12) $$f\left(x\right)=\sum_{i=1}^{N-1}[100{\left(x_{i+1}-x_{i}^{2}\right)}^{2}+{\left(1-x_{i}\right)}^{2}]$$

where x=($x_{1,\ldots ,}x_{N})\in {\mathbb{R}}^{N}$.

The objective function from each variable is contributed, which optimizes the value. The general form for the objective function is expressed as shown in Equation (13)

(13) $$Minimize\;or\;Z\;or\;g_{best}=\sum_{i=1}^{n}c_{i}X_{i}$$

where, $c_{i}$ is known as the objective function coefficient for the ith variable and $X_{i}$ is known as the ith decision variable.

The following Algorithm 1 represents the proposed Hybrid SMO-HPSO algorithm.
**Algorithm 1:** Hybrid SMO-HPSO1:  Input: Intrusion detection feature set and population size 2:  Output: Optimal feature set 3:  Initialize SM Population, Probability, Parameters, Local Leader limit and Global leader limit particles with hierarchy in the population. 4:  Calculate the importance of each feature. 5:  Evaluate the fitness of all SM. 6:  Compute leaders of global and local level leader agents for feature subset. 7:  While iteration I>Maximum iterations 8:   For I=1 to n 9:     Calculate fitness for every particle using Equation (9). 10:     Update particle’s local best position as per hierarchical position. 11:     Based on hierarchical particle velocity update position of all spider monkeys. 12:   End for 13:  Select group members by applying greedy selection method 14:  Update SM’s global optimal position 15:  For I=1 to n 16:   Perform local and global leader learning phase using Equations (5) and (6). 17:   Update particle’s velocity with hierarchy using Equation (2). 18:   Update SM’s global best position. 19:  End for 20:  End while

Therefore, the selection of best set of features from the subset is functioned and if any uncertainty among the features is present, then information gain is evaluated.

### 3.4. Classification Using Random Forest

The best results from the feature selection process are provided to the RF classifier, which uses them to classify the attacks. The decision trees in the RF, an ensemble classifier designed to increase accuracy of the model, operate with smaller classification errors than the models that are currently being used. The number of trees with the smallest node size is also taken into account, and each node is split based on the number of features. The usage of RF classifier in the research work is given as follows:

- The trees are grown and are saved as a future reference.
- The RF classifier overcomes the problem of overfitting.
- The important features developed in the model show improvements in accuracy that are generated automatically to classify the attacks.

Each tree is built from scratch using a bootstrap sample that contains the raw data. Every object that makes up the developed forest is referred to as a tree. Following the values of a tree, decisions are made. In order to classify things, the optimal decision for the tree is made, and every object is then voted on. The class is chosen from the forest with the highest amount of object votes. For the selection of the random variables used to construct the tree, the RF employs both the bagging and boosting processes. The characteristics of RF are as follows:

The generalization error is bound in the RF as it is dependent on the tree strengths among them with a correlation. Preceding the source of maximum voting approach, the maximum number of votes for the RFC is conducted, which classifies the attacks based on Equation (14)

(14) $$prox\left(i,j\right)=\frac{\sum_{t=1}^{ntree}I\;\left(h_{t}\left(i\right)=h_{t}\left(j\right)\right)}{ntree}$$

From Equation (14), $I\left(\cdot \right)$ is known as the indicator function, and $h_{t}$ is tree from forest.

$h_{t}\left(i\right)$ is a predicted value.

If $prox\left(i,j\right)$ = 1 then i and j are of the same classes for classification, the RF provides important ranks or the variables for important feature selection.

## 4. Results and Discussion

The proposed hybrid SMO-HPSO uses Python 3.6 tool with Anaconda Navigator. The windows 10 version with 128 GB RAM running with intel i9 operating at 3GHz and the hardware drive capacity of 4TB is preferable. The following are the performances evaluated for the proposed SMO-HPSO represented by Equations (15)–(22).

Accuracy

(15) $$Accuracy=\frac{\left(TP+TN\right)}{\left(TP+TN+FP+FN\right)}\;$$

Precision

(16) $$Precision=\frac{TP}{TP+FP}$$

Recall

(17) $$Recall=\frac{TP}{TP+FN}$$

F1-Measure

(18) $$F1-measure=\frac{2*Precision*Recall}{Precision+Recall}$$

Error Rate

(19) $$Error\;Rate=\frac{\left|approx-exact\right|}{exact}$$

Area Under the Curve

(20) $$AUC=\int_{a}^{b}f\left(x\right)dx$$

$y=f\left(x\right)$ is the curve equation; where, x=a  and x=b.

False Alarm Rate (FAR)

(21) $$FAR=\frac{FP}{FP+TN}$$

Matthews Correlation Coefficient (MCC)

(22) $$MCC=\frac{TP\times TN-FP\times FN}{\sqrt{\left(TP+FP\right)\left(TP+FN\right)\left(TN+FP\right)\left(TN+FN\right)}}$$

FN—False Negatives.

TP—True positives.

FP—False Positive.

TP—True Positive.

### 4.1. Quantitative Analysis for NSL-KDD Dataset

The results obtained of the proposed Hybrid optimization model in terms of performance are tabulated in Table 2. These outcomes are for binary classification with respect to the NSL-KDD-experiment.

**Figure 5:**
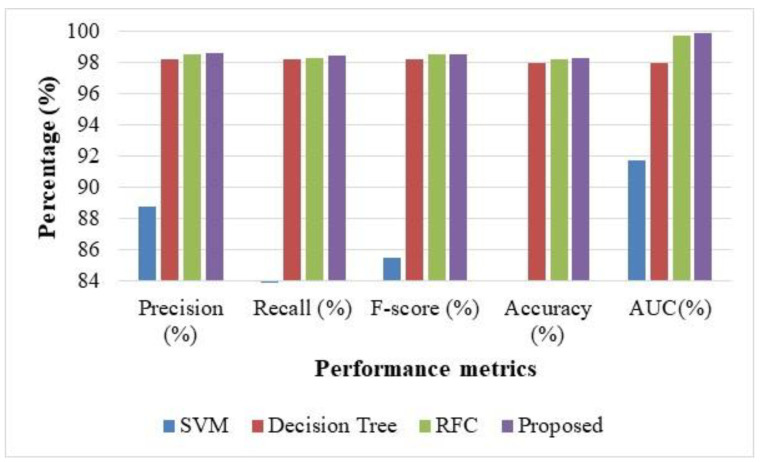
Evaluation of performances for the SVM, DT, RFC, and the proposed method for the NSL-KDD dataset.

Table 2 displays the performance measure values attained for the proposed Hybrid optimization method, which is related to the existing models such as SVM, DT, and RFC. For example, the value of Precision is obtained as 88.81%, 98.22%, 98.56%, and 98.61% for the SVM, DT, RFC, and the proposed model. Figure 5 shows the evaluation of performances for the proposed SMO-HPSO model, SVM, DT, RFC, for NSL-KDD. The overall outcomes presented that proposed hybrid optimization attained superior outcomes when related to traditional methods, which is shown in Table 2.

**Figure 6:**
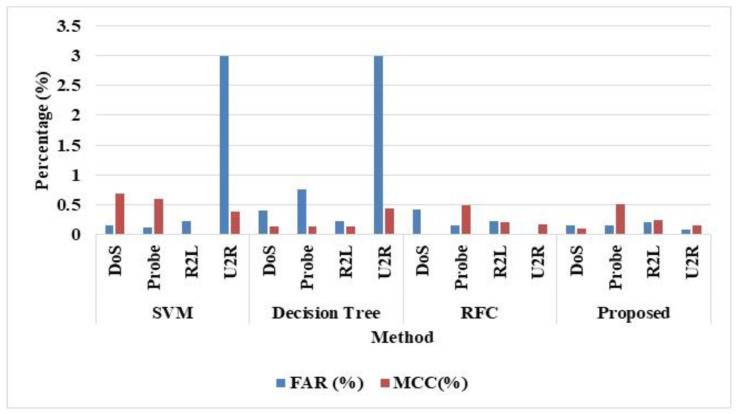
Performance evaluation for the proposed SMO-HPSO for NSL-KDD dataset to multiple attack classes.

In Table 3, we show the multi attacks classification evaluated for the NSL-KDD dataset, which validates results attack types such as DoS, Probe, R2D and U2R. The results are evaluated for the attacks in terms precision, accuracy, F-measure, and recall. The SVM was used to evaluate the results, as it uses the hyper plane parameters to tune, which fails to perform well. The dataset that has more targets overlaps the class with the NSL-KDD dataset. The RF classifier uses the data to consider the feature sets that are irrespective of scales, which shows better performances. The RFC overcomes the multi class problems that are dependent, and it considers the factors of the dataset. For example, the tabulations show that the U2R attack achieves better accuracy of 99.96% values compared with other attacks. Similarly, the DoS attack achieves better precision of 99.89% when compared with other Probe, R2L, and U2R attacks. Figure 6 shows the performance evaluation for SMO-HPSO for NSL-KDD dataset to multiple attack classes.

### 4.2. Quantitative Analysis for UNSW NB-15 Dataset

**Figure 7:**
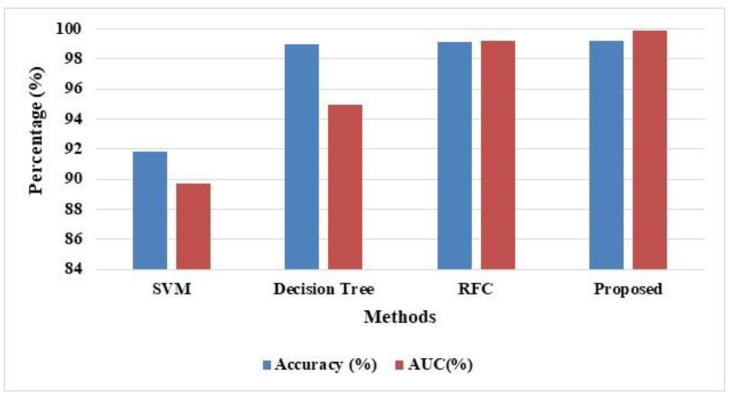
Performance evaluation for the proposed SMO-HPSO for UNSW NB-15 Dataset.

The classification of attacks for overall attacks are evaluated for the UNSW NB-15, which validates the results, as shown in Table 4. The evaluation of the results is in terms of AUC, FAR, and MCC. The SMO-HPSO achieves better results compared to the existing methods SVM, DT, RFC, and SMO-HPSO classifier. The tabulations attack achieves better accuracy of 92% values when compared with other attacks. Similarly, 89% of accuracy was obtained via SVM classifier. The DT has obtained 99% accuracy and AUC of 95%, 99% of accuracy by the RFC classifier and AUC of 95%. The small changes in DT affect the overall structure; therefore, it provides inaccurate results. Similarly, the large number of trees in RFC causes ineffective predictions and reduced accuracy. The SMO-HPSO attained higher accuracy of 99% and AUC of 99.9% values better when compared with other attacks, which is shown in Figure 7.

Similarly, Table 4 displays the performance evaluation metrics for SMO-HPSO for the UNSW NB-15 dataset. The proposed SMO-HPSO obtained better FAR and MCC values of 0.15 and 0.19 compared to existing SVM, DT, and RFC models.

### 4.3. Comparative Analysis

Table 5 shows the comparison results of Hybrid SMO-HPSO with the existing classifiers such as DNN, LSTM-RNN, BAT-MC, ensemble learning, stacking-ensemble technique, optimized deep neural network architecture, LSTM-based CNN, integrated rule based model, and pigeon-inspired optimizer. The existing models utilized a deep learning DBN model to investigate the performance differences during classification [11]. However, the increments in training and testing are not acceptable for all scenarios [12]. Similarly, the BAT algorithm showed the most misclassification instances because they failed to appear often in both the training and test datasets [19]. Similarly, an ensemble-based algorithm was developed that showed effective feature representation and not extracted features [20]. The existing Optimized deep neural network architecture obtained accuracy of 98%, precision of 98%, Recall of 98%, and F-measure 98% as the features vanished and exploded the gradients [26]. LSTM-Based CNN [28] easily over fitted the data, and thus obtained an accuracy of 98.64%. However, the large datasets required time to process the model, which was prolonged to obtain the best performance. This means that the more you train your data on the same points, it will start to treat the noise as data and will just imitate the entire pattern, since it is the same structure dataset used for training and testing and the imbalance in the classes results in an increased detection rate. Similarly, the results are evaluated for the UNSW NB-15 dataset with the existing models, as shown in Table 5. In Kumar et al. [22], an integrated rule-based classifier was used for the classification of attacks. Moreover, feature selection based on the PIO algorithm [25] was used for feature reduction that obtained better accuracy values of 86.9% when compared with the classifier model that obtained 83.8%. The accuracy of the hybrid SMO-HPSO for NSL-KDD dataset outperformed the other existing methods. The F-measure of the hybrid SMO-HPSO was 98.87% for the NSL-KDD dataset, whereas the LSTM-RNN [11] achieved the F-measure of 98.94%. This F-measure analysis shows that the hybrid SMO-HPSO achieves acceptable F-measure with better classification accuracy, even though it is processed with fewer features. The proposed hybrid SMO-HPSO obtained better accuracy of 99.18% for the UNSW NB-15 dataset, which is high when compared to existing models. The classification accuracy for SMO-HPSO increased because of the optimal feature subset selection from overall features. Redundant and irrelevant features that cause the misclassification are avoided by considering the feature importance with the Rosenbrock function as the gradient in the Hybrid SMO-HPSO. The searching process of Hybrid SMO-HPSO for optimal features is improved by combining the hierarchical PSO’s velocity with SMO’s searching process. Therefore, the proposed Hybrid SMO-HPSO provides better classification in the intrusion detection over IoT.

## 5. Conclusions

The present research work performs a hybrid SMO-HPSO algorithm to detect the attack and is classified based on the UNSW NB-15 and NSL-KDD datasets. A hybrid feature selection model is developed using Hybrid SMO-HPSO, which solves the massive intrusion detection on improving the accuracy. The hybrid SMO-HPSO selects the optimal feature subset from overall features based on the incorporation of feature importance with the Rosenbrock function. The searching process of SMO-HPSO is improved by integrating the velocity of hierarchical PSO with SMO’s searching process. Therefore, the developed Hybrid SMO-HPSO enhances the classification accuracy and achieves a lower classification error while classifying the attacks. The SVM model obtained accuracy of 91.82%, DT of 98.99%, and RFC of 99.13%, and the proposed model obtained 99.175% for the NSL-KDD dataset. Similarly, SVM obtained accuracy of 85.88%, DT of 88.87%, RFC of 91.65%, and the proposed model obtained 99.18% for the UNSW-NB 15 dataset. The proposed SMO-HPSO obtained accuracy of 99.175% compared to the existing Deep (DNN) of 97.72%. The Ensemble Learning model showed 85.2% for the NSL-KDD dataset. Additionally, the proposed SMO-HPSO showed average better accuracy of 99.178% and an integrated model that obtained accuracy of 86.9% and 83.8%. However, the soft computing of the hybrid optimization process showed complexity in the system. Yet, identification of the new unknown activities for overcoming the system complexity problem is needed in the future.

## Figures and Tables

**Table 1:** NSL-KDD dataset and UNSW dataset.

NSL-KDD Dataset	Abnormal				Normal	Total
	DoS	Probing	R2L	U2R		
KDD Test+	7458	2754	2421	200	9711	22,544
KDD Train+	45,927	11,656	995	52	67,343	125,973
UNSW Dataset						
Type			No. of Records			
Normal			2,218,761			
Attacks			321,283			

**Table 2:** Performance evaluation for the proposed SMO-HPSO for NSL-KDD dataset to binary attack classes.

Methods	Precision (%)	Recall (%)	F-Score (%)	Accuracy (%)	FAR (%)	AUC (%)	MCC (%)
SVM	88.81	82.41	85.48	84.06	0.28	91.70	0.51
Decision Tree	98.22	98.18	98.21	97.95	0.45	97.96	0.22
RFC	98.56	98.27	98.49	98.17	0.21	99.68	0.61
Proposed	98.61	98.41	98.56	98.31	0.21	99.87	0.17

**Table 3:** Performance evaluation for the proposed SMO-HPSO for NSL-KDD dataset to multiple attack classes.

Methods	Class	Precision (%)	Recall (%)	F1 Measure (%)	Accuracy (%)	AUC (%)
SVM	DoS	97.44	82.88	89.56	91.61	97.17
	Probe	87.62	91.48	89.33	92.82	97.1
	R2L	89.31	64.09	67.11	83.37	67.2
	U2R	89.2	72.2	77.27	99.51	97.37
DT	DoS	99.46	99.67	99.57	99.63	99.63
	Probe	98.83	98.72	98.77	99.22	98.69
	R2L	96.79	96.24	96.51	97.55	97.58
	U2R	86.77	83.71	84.41	99.58	83.71
RFC	DoS	99.86	99.71	99.82	99.81	99.96
	Probe	98.92	98.71	98.81	99.26	99.67
	R2L	97.26	96.55	96.79	97.77	99.41
	U2R	96.64	85.21	89.13	99.7	97.62
Proposed	DoS	99.78	99.66	99.72	99.75	99.98
	Probe	99.34	99.3	99.29	99.34	99.89
	R2L	97.19	96.64	96.69	97.85	99.66
	U2R	99.75	97.74	99.78	99.76	99.87

**Table 4:** Performance evaluation for the proposed SMO-HPSO for UNSW NB-15 Dataset.

Methods	Precision (%)	Recall (%)	F-Score (%)	Accuracy (%)	FAR (%)	AUC (%)	MCC (%)
SVM	89.36	85.93	88.73	85.88	0.88	94.25	0.43
Decision Tree	90.77	88.85	90.40	88.87	1.10	95.53	0.11
RFC	92.57	91.33	91.84	91.65	0.21	98.44	0.22
Proposed	94.19	93.32	94.12	99.18	0.15	99.78	0.19

**Table 5:** Comparative analysis.

Authors	Dataset	Method	Accuracy (%)	Precision (%)	Recall (%)	F-Measure (%)
Elmasry et al. [11]	NSL-KDD	DNN	97.72	99.6	96.38	97.96
		LSTM-RNN	98.8	99.7	98.18	98.94
Su et al. [12]		BAT-MC	84.25	-	-	-
Gao et al. [19]		Ensemble Learning	85.2	86.5	85.2	84.9
Çavuşoğlu [20]		Stacking-ensemble technique	99.78	90	-	92.68
Ramaiah et al. [23]		Optimized Deep Neural Network Architecture	98	98	98	98
Hsu et al. [25]		LSTM-based CNN	98.64	-	-	-
Kumar et al. [22]	UNSW NB-15	Integrated rule based Model	83.8	-	-	87.95
Alazzam et al. [28]		Pigeon-Inspired Optimizer	86.9	-	86.6	86.4
Proposed Method	NSL-KDD	SMO-HPSO	99.175	99.015	98.335	98.87
	UNSW NB-15		99.18	94.19	93.32	94.12

## Data Availability

The datasets generated during and/or analyzed during the current study are available in the [UNSW-NB 15 datasets] and [NSL-KDD datasets] repositories, UNSW-NB 15 datasets available link: https://www.unsw.adfa.edu.au/unsw-canberra-cyber/cybersecurity/ADFA-NB15-Datasets/, accessed on 12 August 2022. NSL-KDD datasets available link: https://www.unb.ca/cic/datasets/nsl.html, accessed on 12 August 2022.
